# Treadmill Exercise Impact on Brain Electrophysiological and Glial Immunoreactivity in Cuprizone-Treated Rats

**DOI:** 10.3390/brainsci15070686

**Published:** 2025-06-26

**Authors:** Cássia Borges Lima-de-Castro, Noranege Epifânio Accioly, Geórgia de Sousa Ferreira Soares, Catarina Nicácio dos-Santos, Sonia Carolina Guerrero Prieto, Rubem Carlos Araujo Guedes

**Affiliations:** Department of Nutrition, Universidade Federal de Pernambuco, Recife 50670901, Brazil; cassiacastro1975@gmail.com (C.B.L.-d.-C.); noranege@hotmail.com (N.E.A.); georgiasousafs@gmail.com (G.d.S.F.S.); catarina.nicacio@ufpe.br (C.N.d.-S.); sonicdc30@gmail.com (S.C.G.P.)

**Keywords:** brain development, cuprizone, demyelination, electrical activity, glial cells, physical exercise

## Abstract

**Background/Objectives**: Demyelination occurs to a variable extent in various neurological diseases, such as multiple sclerosis. Physical exercise benefits central neural functions that depend on the brain’s electrophysiological and glial activity. It is unclear whether both factors—i.e., demyelination and exercise—interact in the brain. We aimed to investigate if this interaction occurs during brain development. **Methods:** Developing rats were subjected to a cuprizone-induced demyelination. Part of these rats were treadmill-exercised for five weeks. After this period, some demyelinated animals were allowed to remyelinate by receiving a similar diet, without cuprizone, for six weeks. The exercised groups were compared with the corresponding sedentary groups. All groups were evaluated electrophysiologically (cortical spreading depression features), and their brains were processed for immunohistochemical labeling with four specific glial antibodies (anti-APC, MBP, GFAP, and Iba1). **Results:** Compared with the corresponding controls, cuprizone demyelination and treadmill exercise accelerated and decelerated CSD propagation. Cuprizone reduced APC, MBP, and GFAP immunolabeling and increased Iba1 immunostaining. Remyelination reverted the cuprizone effects. Exercise counteracted the cuprizone-induced changes in GFAP- and Iba1-containing cells but not in MBP- and APC-containing ones. **Conclusions:** Our data confirmed the effectiveness of the cuprizone demyelination paradigm. They evidenced the potential neuroprotective effect of regular physical exercise, suggesting that this non-pharmacological intervention could benefit patients with central demyelination-dependent diseases.

## 1. Introduction

Demyelinating diseases (DDS) are chronic pathological processes resulting from complex interactions between genetic and environmental factors, involving the immune system [1,2]. One of the most widely investigated DDS is multiple sclerosis (MS), characterized by inflammation, demyelination, neurodegeneration, and death of glial cells that occur to a variable extent depending on the intensity and reversibility of the demyelination process [3,4]. Because MS is a chronic degenerative disease, patients often live with it for most of their useful life. Physical activity appears to aid in attenuating multiple sclerosis symptoms [5].

To study the complex mechanisms of Dds, several animal models, including toxic models of central demyelination, have been employed [6,7,8]. In the cuprizone toxicity model, young rodents are fed a diet containing cuprizone, a copper chelator, which causes the death of oligodendrocytes and the activation of microglia [9]. The cuprizone demyelination is reversible: upon withdrawal of the cuprizone, spontaneous remyelination occurs [10]. In rodents, cuprizone provokes demyelination in the brain’s white and gray matter, as well as in the cerebellum, but does not affect the peripheral nervous system [9,11]. While most experimental studies using cuprizone are performed in mice, we aimed to investigate such effects in the rat brain. In agreement with the observations of Zhen and co-workers [12], our data demonstrate cuprizone effects on CSD and glial immunoreactivity that are like those observed in mice [10]. Apoptosis of primary oligodendrocytes in conjunction with microglial activation is a major histopathological feature of the animal cuprizone demyelination model. This pathological feature is also found in the formation of MS lesions in humans [13]. Demyelinating conditions of the central nervous system constitute one of the primary causes of several debilitating neurological diseases, including those associated with aging.

In patients with MS, physical exercise has been shown to produce beneficial therapeutic effects [3]. On the other hand, in laboratory animals, regular physical exercise benefits the nervous systems of young, adult, and elderly individuals [14]. Physical exercise enhances the performance of tasks that involve brain electrophysiological functions [15] and cognitive performance [16], as well as to mitigate the neural effects of aging [16,17]. In the brains of animals, exercise is associated with neurogenesis and increased neuronal survival [18,19,20]. Exercise also increases vascular blood flow in the cerebellum and motor cortex [21,22,23]. Physical exercise on the treadmill at the beginning of life influences cerebral electrical activity, as evidenced by analyzing the propagation of the phenomenon known as cortical spreading depression [CSD] [24].

CSD, a reversible, propagating wave of reduction in spontaneous cortical activity, occurs due to brain cell depolarization following the stimulation of a single cortical point with electrical, mechanical, or chemical agents. Once initiated in the stimulated cortical area, CSD spreads slowly across the tissue [25]. CSD has been postulated as participating in several human neurological diseases, such as epilepsy, migraine, traumatic brain injury, and subarachnoid hemorrhage [26]. Studies on animals have revealed that CSD propagation can be accelerated or decelerated through hormonal, pharmacological, environmental, and nutritional manipulations [27]. The animal studies compellingly suggested that the analysis of CSD velocity of propagation is a useful index that can help comprehend excitability-related processes underlying brain functioning [28].

While, on the one hand, there is still a lack of effective neuroprotective drugs designed to delay neurodegenerative processes, on the other hand, numerous reports suggest that regular physical activity could potentially reduce the risk of neurological impairment in conditions such as MS, stroke, and Parkinson’s disease [29,30,31,32,33]. Furthermore, evidence of exercise-induced transcription enhancement of neurotrophic factors has been reported [14]. Therefore, the effects of physical exercise are shown to be neuroprotective, promote brain health, precondition the brain against ischemic insult, and improve its long-term functioning [34]. Although pharmacological approaches are making great advances, evidence indicates that non-pharmacological approaches, such as physical exercise, can promote anti-inflammatory and neuroprotective actions in patients with MS [35].

Given that the brain’s electrical activity controls its primary functions, studying the CSD electrophysiological phenomenon represents a crucial tool for understanding brain functioning and neuronal signaling in both health and disease [36]. Our laboratory has considerable experience in CSD studies relating this phenomenon to several nutritional and non-nutritional variables (see [36]). In cuprizone-treated mice, the CSD features have been investigated by Merkler and co-workers [10]. In the current study, we extended the investigation of mice by examining in rats the effects, in adult life, of the association between cuprizone and treadmill exercise early in life.

This study aimed to examine the long-term effects of treadmill exercise, with or without cuprizone-induced demyelination, on the developing rat brain, evaluated later in adulthood. We assessed changes in the electrophysiological features of cortical spreading depression (CSD) and the immunolabeling patterns of glial reactions in both exercised and sedentary rats. We compared the parameters in normally myelinated (control) rats to those in animals subjected to a cuprizone diet that induced demyelination. We hypothesized that treadmill exercise early in life would attenuate, in adult life, the brain’s ability to propagate CSD and the glial responses in cuprizone-induced demyelinated rats.

## 2. Materials and Methods

### 2.1. Animals

The animals (Wistar rats) were handled following the norms of the Ethics Committee for Animal Research of our University (Approval protocol no. 23076.019381/2014-26; approved on 16 September 2014), which complies with the “Principles of Laboratory Animal Care” (National Institutes of Health, Bethesda, MD, USA). Groups of 2–3 rats were housed in polypropylene cages (51 × 35.5 × 18.5 cm) in a room maintained at 23 ± 1 °C with a 12:12 h light-dark cycle (lights on at 7:00 a.m.) and had free access to a lab chow diet with 23% protein.

### 2.2. Cuprizone Administration

From postnatal day 28 (P28) to P62, rats were fed either the control diet (ConD; n = 19) or the cuprizone diet (CupD; n = 39), which contained 0.2% (*w*/*w*) cuprizone (Sigma, St. Louis, MO, USA). We used cuprizone treatment as a model of toxic demyelination and remyelination [10]. As described below (Section 2.3), both exercised (n = 30) and sedentary animals (n = 28) were subdivided into three treatment conditions, forming the six groups in this study: two groups received the control diet (9 exercised and 10 sedentary rats; respectively, groups no. 1 and 2); two groups received the demyelinating (cuprizone-based) diet (11 exercised and 10 sedentary rats; respectively, groups no. 3 and 4); and two groups, after demyelination, were switched back to the control diet to experience remyelination for 6 weeks (10 exercised and eight sedentary rats; respectively, groups no. 5 and 6). These six groups are presented in Table 1.

### 2.3. Treadmill Exercise

Moderate exercise was conducted on a motorized treadmill apparatus (Insight EP-131, 0° inclination), as previously described [24]. Over 7 weeks, one daily session of exercise (30 min/day) was performed on a 5-day/week basis, followed by a 2-day rest. The exercise was initiated on postnatal day 28 (P28) and terminated on P62 immediately before the CSD recording day. The treadmill running velocity increased from 5 m/min during the first training week to 10 m/min during the second week and further increased to 15 m/min from the third week to the end of the exercise period. The sedentary groups remained on the treadmill for the same period as the exercised animals; however, the treadmill was kept off. The apparatus contained six running tracks. Each track was 10 cm wide and 40 cm long; therefore, there was not much space for the rat to walk, except when the treadmill was on. The body weights were measured on P30 and P60.

### 2.4. CSD Recording

**Figure 1:**
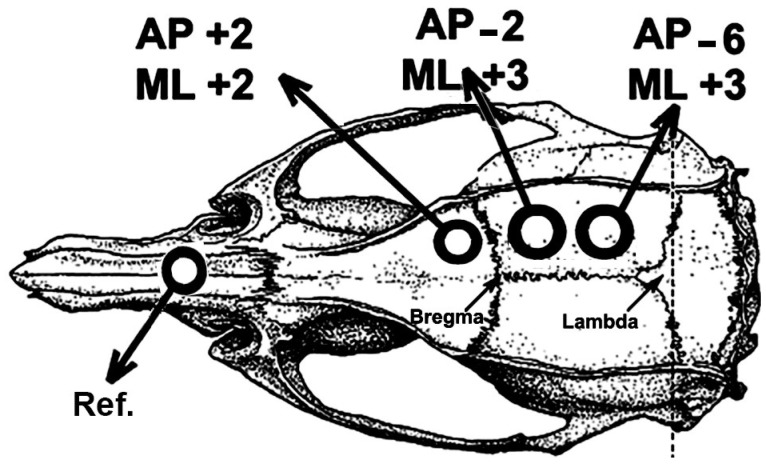
Drawing of the rat skull, showing the positions of the three trephine holes drilled on the right side of the skull. The AP and ML coordinates are set according to the rat stereotaxic atlas of Paxinos and Watson [37]. The position of the reference electrode (Ref.) is indicated on the nasal bones.

After the treadmill exercise and the demyelinating and re-myelinating dietary paradigms, rats were subjected to a CSD recording session for 4 h, as previously described [24]. Under anesthesia (1 g/kg urethane plus 40 mg/kg chloralose, i.p.), the rat’s head was secured into a stereotaxic apparatus (Kopf, Miami, FL, USA), and three trephine holes (2–3 mm in diameter) were drilled on the right side of the skull. These holes—one at the frontal bone and two on the parietal bone—were aligned in the anterior-to-posterior direction and parallel to the midline. The center of the holes was set at the following stereotaxic coordinates (Figure 1) according to the stereotaxic atlas of Paxinos and Watson [37]: AP +2/ML +2, AP −2/ML +3, and AP −6/ML +3. CSD episodes were elicited at 20-min intervals by applying, for 1 min, a cotton ball (1–2 mm diameter) soaked in 2% KCl solution (approximately 0.27 M) to the anterior (frontal) hole.

The slow direct-current (DC) potential change, which is the hallmark of CSD, was recorded at the two parietal points on the cortical surface using a pair of Ag-AgCl agar-Ringer electrodes. These electrodes consisted of plastic conical pipettes (5 cm in length, with an inner diameter of 0.5 mm at the tip) filled with Ringer solution, solidified by adding 0.5% agar, into which a chlorided silver wire was inserted. A pair of such pipettes was fixed with cyanoacrylate glue, ensuring that the interelectrode distance for each pair remained constant (the distance for different pairs ranged from 4 to 6 mm). The electrode pair was attached to the electrode holder of the stereotaxic apparatus, allowing the electrode tips to be gently placed on the intact dura mater under low-power microscope control without excessive pressure on the cortical surface. A common reference electrode of the same type was placed on the nasal bones (Figure 1). The CSD parameters (propagation velocity, amplitude, and duration of the negative DC-potential change) were measured as previously reported [38]. CSD velocity was calculated based on the time required for a CSD wave to cross the interelectrode distance. In measuring CSD parameters, the initial point of each DC negative rising phase was used as the reference point. During the recording session, rectal temperature was maintained at 37 ± 1 °C using a heating blanket. At the end of the recording session, some of the animals were subjected to perfusion-fixation procedures for subsequent immunohistochemical analysis (see Section 2.5 below): trans-cardiac perfusion was performed with phosphate-buffered saline (pH 7.4), followed by 0.4% paraformaldehyde fixation.

### 2.5. Immunohistochemical Analysis of Glial Cells

Considering that the CSD phenomenon occurs in the cortex grey matter, we decided to immuno-label the cortex. A total of 11 sedentary rats (groups 2, 4, and 6; n = 4, 3, and 4, respectively) and 11 exercised animals (groups 1, 3, and 5; n = 4, 3, and 4, respectively) were perfused, as described above. After being removed from the skull and immersed into the fixative for four hours, the brains were transferred to a 30% (*w*/*v*) sucrose solution for cryoprotection. Longitudinal serial sections (40 µm thickness) were obtained at −20 °C with a cryo-slicer (Leica 1850, Wetzlar, Germany). Four series of sections were immunolabeled, respectively: (1) with mouse anti-APC to detect oligodendrocytes (anti-APC, #OP62; Calbiochem, Burlington, MA, USA); (2) with rat anti-myelin basic protein (MBP) antibody to detect myelin (anti-MBP, #MAB386, Millipore, Burlington, MA, USA); (3) with a polyclonal rabbit antibody against glial Fibrillary Acidic Protein (GFAP) to detect mature astrocytes (anti-GFAP, #Z0334; Dako, Glostrup, Denmark); (4) with a polyclonal antibody against the ionized calcium-binding adapter molecule 1 (Iba1) to detect microglia (anti-Iba1, #019-19741; Wako Pure Chemical Industries Ltd., Osaka, Japan). Free-floating, Tris-buffered saline (TBS)-washed (3 times for 5 min) sections were submitted to endogenous peroxidase blocking (2% H_2_O_2_ in 70% methanol for 10 min); then, sections were incubated for one h in Blocking Buffer (BB) solution containing 0.05 M (TBS) pH 7.4, 10% fetal calf serum, 3% bovine serum albumin, and 1% Triton X-100. Afterward, sections were incubated overnight at 4 °C with primary antibodies against rat anti-APC (1:250 diluted in BB solution) to detect oligodendrocytes, mouse anti-MBP (1:1000 diluted in BB solution) to detect myelin, rabbit anti-GFAP (1:2400 diluted in BB solution) to detect GFAP, and rabbit anti-Iba1 (1:3000 diluted in BB solution) to detect microglia. After three washes with TBS + 1% Triton, sections were incubated at room temperature for 1 h with biotinylated secondary antibodies: rat anti-APC (1:500 diluted in BB solution) to detect oligodendrocytes, mouse anti-MBP (1:200 diluted in BB solution) to detect myelin, rabbit anti-GFAP (1:500 diluted in BB solution) to detect astrocytes, and rabbit anti-Iba1 (1:500 diluted in BB solution) to detect microglia. Sections were then rinsed in TBS + 1% Triton and incubated with horseradish peroxidase streptavidin (1:500). The peroxidase reaction was visualized by incubating the sections in Tris buffer containing 0.5 mg/mL 3, 3′-diaminobenzidine (DAB), and 0.33 μL/mL H_2_O_2_. Sections were finally mounted, dehydrated in graded alcohols, and, after xylene treatment, coverslipped in Entellan^®^ (Merck, Darmstadt, Germany) For each animal, densitometric analysis was performed on four parallel longitudinal brain sections. In each section, we analyzed photomicrographs of four fields within layer 5 of the motor cortex (AP: +1.0 to −1.0; ML: 1.90 to 2.62 mm) [37] using ImageJ software (National Institutes of Health, USA, version 1.46r). A Leica DMLS microscope coupled to a Samsung high-level color camera (model SHC-410NAD) was used to obtain digital images from brain sections. Images from the immunoreacted motor cortexes were obtained with a 20× microscope objective. Care was taken to obtain the digital images using the same light-intensity conditions. We analyzed the percentage of the area occupied by the immuno-labeled cells and their total immunoreactivity expressed as arbitrary units. As described previously by Francisco and co-workers [39], “the color images were first converted into a grayscale. Based on the color difference, the algorithm in the program identified the darker areas (marked cells) in relation to the lighter areas (background), and the total marked area was calculated. The threshold for selection was manually adjusted such that the background was not marked. All sections photographed at the same magnification displayed the same total area in the photographs; therefore, the ratios between the labeled cells’ areas and the total picture area could be directly compared. The labeled area was expressed as a percentage of the total area in the picture. The immunoreactivity intensity was obtained in the program by calculating the mean gray value (MGV) within the selected area. The MGV can vary numerically from 0 (darkest) to 255 (lightest). Therefore, the reactivity intensity was given by the difference (255-MGV). By multiplying this value by the marked area, we arrived at a figure (in arbitrary units) that indicated the proportion of the gray area in the image that was due to cell labeling. Mathematically, the more intense the labeling, the greater the arbitrary unit value.” We analyzed the total immunoreactivity and the area occupied by the immunolabeled cells (expressed as percentage of the total area), as previously analyzed [39].

**Figure 2:**
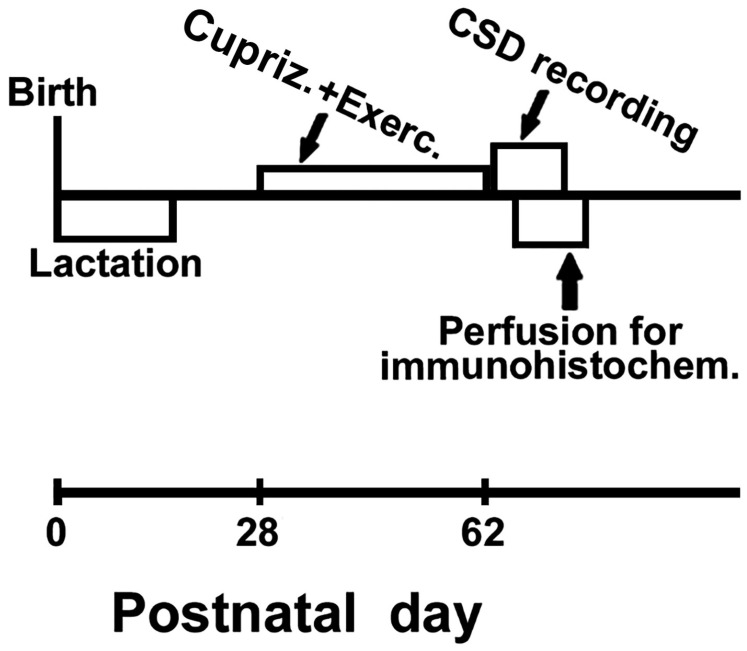
Diagram showing the experimental procedures and the corresponding age ranges (postnatal days) in which they occurred.

A negative control section was included for each immunostaining reaction. In this section, the primary antibody was replaced with Tris-buffered saline (TBS). All negative control sections showed no specific staining. Figure 2 presents the procedures conducted during the experiment, along with the age range in which each procedure occurred.

### 2.6. Statistics

Intergroup differences were compared by ANOVA or Kruskal–Wallis analysis of variance on ranks, followed by a post hoc test when indicated. The statistical software used was “Sigmastat^®^” version 3.5. Differences were considered significant when *p* ≤ 0.05.

## 3. Results

### 3.1. Body Weight

At P30, both sedentary and exercised animals that were treated with cuprizone displayed lower weights than the corresponding cuprizone-free groups (F _[1, 36]_ = 49.339; *p* < 0.001). Such weight reduction persisted at P60 (F _[1, 36]_ = 90.315; *p* < 0.001). Furthermore, at P60, the exercised groups showed a weight reduction compared to the corresponding sedentary groups (F _[1, 36]_ = 36.089; *p* < 0.001). The mean ± SD body weights are in the Table 2.

### 3.2. CSD Features

**Figure 3:**
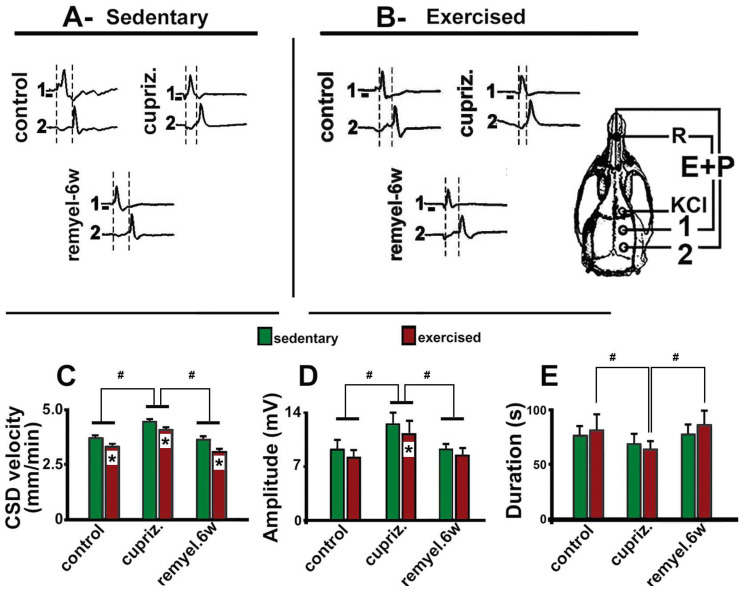
Parts A and B of this figure illustrate examples of CSD recordings in three sedentary (**A**) and three exercised rats (**B**) that are representative of the distinct treatment groups of this study: control (group that received the normal, cuprizone-free diet); demyelinated (group that received the cuprizone diet); and remyelinated (which, after demyelination, was switched to the normal diet for six weeks). The skull diagram shows the two cortical recording positions (1 and 2), from which the traces marked on the left side with the same numbers were obtained. The position of the common reference electrode (R) on the nasal bones and the CSD-eliciting stimulus (KCl) application point are also shown. CSD was elicited on the frontal cortex by 1-min KCl stimulation (with a 1–2 mm diameter cotton ball embedded in 2% KCl) applied on the intact dura mater, as indicated by the horizontal bars under the traces at the recording point 1 at parts (**A**,**B**) of the figure. In these parts (**A**,**B**), the vertical dashed lines indicate the latency for a CSD wave to cross the interelectrode distance. One can note the shorter latencies in the cuprizone group compared to the controls. Parts (**C**–**E**), present CSD velocity, amplitude, and duration (mean ± standard deviation) among the dietary treatment groups. Exercised groups marked with an asterisk differ (*p* < 0.05) from the corresponding sedentary group. The symbol # indicates intergroup significant differences (*p* < 0.05; ANOVA followed by a post-hoc test).

Typical electrophysiological recordings of CSD (slow DC potential change) are shown in Figure 3, parts A and B. These recordings are from three sedentary (Figure 3A) and three exercised rats (Figure 3B), representing the three different dietary treatments: one rat from the control group, one from the cuprizone group, and one from the re-myelinated group. The 1-min stimulation with 2% KCl at one point on the frontal cortex usually elicited a single CSD episode that propagated without interruption and was recorded by the two electrodes located more posteriorly on the surface of the parietal cortex (see stimulation and recording points in the skull diagram of Figure 3). In each recording point, the appearance of the slow potential change confirmed the presence of CSD after KCl application. As a rule, the electrophysiological changes caused by CSD always recovered after a few minutes. We maintained a 20-min interval between subsequent KCl stimulations over the 4 h recording session, as mentioned in the Methods section (Section 2.4 above).

ANOVA revealed that both exercise (F _[1, 52]_ = 176.596; *p* < 0.001) and cuprizone treatment (F _[2, 52]_ = 277.867; *p* < 0.001) affected CSD propagation; no interaction between these two factors was observed (F _[3, 52]_ = 1.869; *p* = 0.165). In the sedentary condition, the Holm–Sidak test indicated that cuprizone treatment was associated with increased CSD velocity (4.4 ± 0.2 mm/min) compared with the group fed the control diet (3.7 ± 0.1 mm/min; *p* < 0.001). This effect was reversed in the remyelinated animals that, after cuprizone treatment, returned to the normal diet (without cuprizone) for 6 weeks (CSD velocities of 3.6 ± 0.1 mm/min). Compared with those velocities, treadmill exercise reduced the propagation rate of CSD (*p* < 0.005) in all groups (3.3 ± 0.2 mm/min in the control group, 4.0 ± 0.1 mm/min in the demyelinated group, and 3.0 ± 0.1 mm/min in the re-myelinated group). Data are presented in the bar graph of Figure 3, part C.

The amplitude and duration of the negative slow potential shift, a hallmark of CSD, are illustrated in Figure 3, part D and Figure 3, part E, respectively. Regarding amplitude, ANOVA revealed the main effect of exercise (F _[1, 52]_ = 8.884; *p* < 0.005) and cuprizone treatment (F _[2, 52]_ = 24.671; *p* < 0.001); no interaction between the two factors (exercise and cuprizone treatment) was observed (F _[3, 52]_ = 0.237; *p* = 0.790). The post hoc test indicated that exercised rats presented with a decreased (*p* < 0.05) CSD amplitude (8.1 ± 0.9 mV) compared with the sedentary group (9.5 ± 1.8 mV). Cuprizone-treated animals presented with an increased (*p* < 0.01) amplitude (12.4 ± 1.9 mV) in comparison with the control rats (9.5 ± 1.8 mV) and the remyelinated group (9.1 ± 0.8 mV). Regarding CSD duration, ANOVA revealed no significant intergroup difference among sedentary animals (F _[1, 52]_ = 0.372; *p* = 0.544). In the exercised rats, cuprizone treatment was associated with shorter CSD duration (F _[2, 52]_ = 8.101; *p* < 0.001).

### 3.3. Immunohistochemistry and Densitometric Analysis

**Figure 4:**
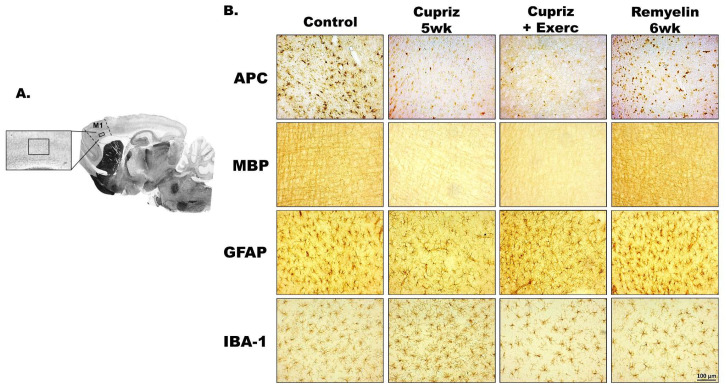
(**A**) Low magnification parasagittal rat brain section illustrating the site (layer 5) in the motor cortex (AP = 0; ML = 2.1), from which the high magnification photomicrographs in B have been obtained. (**B**) Photomicrographs of immunolabeled APC, MBP, GFAP, and Iba1-positive microglial cells in the cortex of 63–66-day-old male rats previously fed a cuprizone-free diet (control group; first column from the left) or a cuprizone-containing diet (cuprizone group; second column from the left). The cuprizone and exercise treatments (third column from the left) and the remyelination paradigm (fourth column from the left) attenuated the effects of cuprizone. The group description is as in Table 1. Scale bar = 100 µm for all photomicrographs.

Figure 4 (photomicrographs) and 5 (bar graphics) show the effect of dietary administration of cuprizone and exercise on the immunolabeled APC, MBP, GFAP, and Iba1-positive cells within the motor cortex.

The Kruskal–Wallis one-way ANOVA revealed that the cuprizone treatment was associated with a decreased immunoreactivity for APC (H = 119.517 with 3 degrees of freedom; *p* < 0.001), MBP (H = 142.707 with 3 degrees of freedom; *p* < 0.001), and GFAP (H = 117.111 with 3 degrees of freedom; *p* ≤ 0.001) and an increased immunoreactivity for Iba1-containing microglia (H = 83.812 with 3 degrees of freedom; *p* < 0.001) (Figure 3 and Figure 4). Microglial cells showed an increased immunoreactivity pattern in the cuprizone-treated groups.

**Figure 5:**
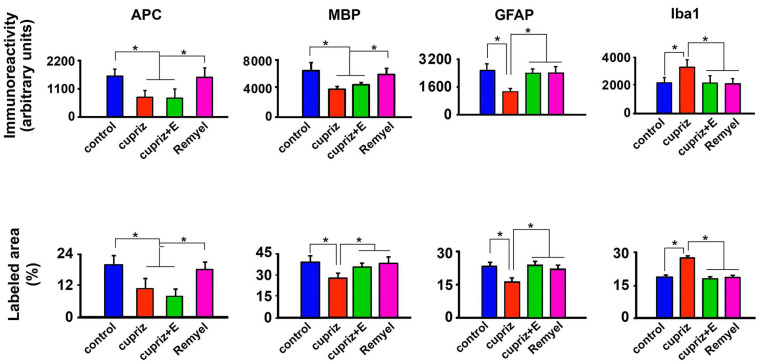
Immunoreactivity (upper half of the figure) and labeled area (lower half) of APC, MBP, GFAP, and Iba1-reactive cells in the motor cortex of four groups of rats named as follows: (1) control group (non-exercised, non-treated with cuprizone; n = 4); (2) cuprizone group (sedentary, treated with cuprizone; n = 3); (3) cuprizone + E group (treated with cuprizone and exercised; n = 3); and (4) remyelination group (cuprizone-treated and then switched to the cuprizone-free diet to allow for remyelination; n = 4). Values are expressed as mean ± standard deviation. The asterisks indicate intergroup differences (*p* < 0.05; Kruskal–Wallis ANOVA followed by Dunn’s test).

Regarding the percentage of labeled areas, the oligodendrocyte labeling (H = 138.911 with 3 degrees of freedom; *p* < 0.001), the myelin labeled area (H = 107.105 with 3 degrees of freedom; *p* < 0.001), and the GFAP (H = 106.800 with 3 degrees of freedom; *p* ≤ 0.001) were significantly decreased in the groups treated with cuprizone compared with the control group. In contrast, cuprizone treatment was associated with an increased Iba1-labeled area (H = 106.458 with 3 degrees of freedom; *p* < 0.001). These data are in Figure 5.

## 4. Discussion

The cuprizone-induced demyelination model and various physical exercise paradigms are widely used to experimentally study pathological processes in the nervous system. The cuprizone model, for instance, is a well-established tool for inducing reversible demyelination in rodents, mimicking certain aspects of multiple sclerosis in humans [40]. Similarly, physical exercise has neuroprotective effects and can positively influence various physiological processes in the brain [14]. However, associating the two paradigms in a single study is not a common practice. Our study was the first to pioneer the association between both models (cuprizone and exercise) and confirmed the deleterious effect of cuprizone on rat brains, which was attenuated by treadmill exercise early in life. The effects of cuprizone and exercise could be demonstrated via electrophysiological measurements (CSD propagation) and glial immunohistochemical analysis.

The reduced body weight in the cuprizone-treated and exercised animals aligns with previous studies [12,24]. The present results are also consistent with studies suggesting that cortical myelin plays a crucial role in determining the velocity of CSD propagation [10]. We observed that cuprizone-demyelinated rats exhibited accelerated CSD propagation, suggesting an inverse correlation between cortical myelin and CSD propagation velocity. In support of this observation, demyelinated animals that were remyelinated (by further feeding again a cuprizone-free diet) presented with CSD velocities comparable to those of the control animals. In addition, our CSD propagation data support the previous suggestion that cortical myelin is involved in ion homeostasis in the cerebral cortex [10].

Furthermore, the observation that the remyelinated animals presented with normal (control-like) CSD velocities reinforces the role of myelin in CSD propagation rather than an indirect action of myelin. The CSD modulation by myelin is dichotomous: transgenic mice expressing hypermyelinated brains displayed reduced CSD propagation velocity [10], which supports the assumption that CSD propagation is inversely influenced by cortical myelin content. Interestingly, studies in early malnourished rats have demonstrated that the malnourished brain propagates CSD significantly faster than the well-nourished control [38]. Additionally, malnourished animals have been found to have lower myelin content compared to well-nourished animals [41]. It is worth noting that, in contrast, rats overnourished during suckling (those from small litters with only three pups) exhibited decelerated CSD propagation [42]. Such evidence also supports the involvement of the brain’s myelin content in the propagation of CSD. Nevertheless, other nutrition-associated factors, such as neurotransmitter activity, cell packing density, or neuron-to-glia ratio, also need to be considered, as previously discussed [10].

The development and maintenance of neural functions depend largely on the physiological activity of glial cells. While oligodendrocytes are the cells responsible for myelination, microglial cells are involved in immune actions in the brain, thereby behaving as immune cells of the nervous tissue [43,44]. In our experiments, cuprizone significantly reduced the numbers of oligodendrocytes, MBP, and GFAP immunolabeling and increased Iba1 immunoreactivity. Oligodendrocytes and MBP reduction are expressions of the cuprizone-induced demyelination, while increased Iba1 immunolabeling indicates the toxic effect of cuprizone. Treadmill exercise reversed the cuprizone effect on Iba1 immunolabeling and had a less intense action on cuprizone-induced demyelination (Figure 5), suggesting that regular exercise practice may be beneficial in at least attenuating the symptoms of demyelinating pathological processes. Nevertheless, further clinical and experimental investigations are needed to confirm our suggestion.

The reduced glial cell activity is a crucial factor in our CSD findings, as evidence suggests that glial cells play a role in cortical resistance to CSD propagation [45]. Additionally, the current data on APC and MBP immunolabeling confirmed the occurrence of demyelination and remyelination processes, corroborating previous investigations [4,10,46]. Exercised mice presented with plastic alterations in the brain, such as complex tree planting, that correlated with motor functions [47]. These alterations lead us to suggest that the neural plasticity caused by exercise can attenuate the toxic effects of cuprizone.

Our studies have evidenced the importance of physical exercise and its potential neuroprotective effects as a non-pharmacological intervention in pathologies where demyelination occurs. Studies in rodents and humans indicate that physical exercise improves both cognitive and motor functions in neurodegenerative models for stroke [48], Parkinson’s disease, Alzheimer’s disease, and multiple sclerosis [33,49,50]. In a mouse study, exercise was found to increase oligodendrogenesis in the intact spinal cord [51]. Another relevant factor of our study is that myelin presented a lower immunoreactivity pattern in the groups treated with cuprizone. This lower immunoreactivity would be expected because the cells responsible for myelinization of the central nervous system were reduced. In a study on mice, exercise improved neuromuscular function and induced early protection against axonal damage and loss of proteins associated with myelin in the striatum and corpus callosum, in addition to attenuating microglial activation in these brain regions [52]. Preclinical studies suggest that exercise may involve multiple immunoregulatory mechanisms, likely due to attenuation of the T-cell response and brain infiltration. However, direct neurotrophin-mediated neuroprotective mechanisms are likely involved [53].

## 5. Limitations of Our Study

### 5.1. Animal’s Sex

Our study examined only male rats; this limitation was motivated by the rapid and complex hormonal oscillations in female rats, which can influence behavioral and brain CSD features [54] and impact the cuprizone effect [55]. We recognize the need to extend our observations to the female rat. However, it is a common strategy to concentrate initial efforts on studying male rats and then, with the male outcome as a well-established database, investigate female rats using the same methodologies.

### 5.2. Animal Species

Whether the demyelination/exercise interaction demonstrated here can be extrapolated from rats to other mammalian species and the developing human brain is unknown. At least among the two most used rodent species, rat and mouse, the effects of cuprizone regarding reversible demyelination are similar [12]. Our experiments add information about using the cuprizone model in the rat brain. If the extrapolation from rats to humans could be confirmed, the evidence suggests a possible relevant implication of our novel findings in the developing rat brain. Nevertheless, we recognize the importance of replicating our findings in mammal species other than the rat to strengthen our conclusions. However, the lack of such additional studies does not invalidate our results; rather, it makes them more interesting.

### 5.3. Routes of Cuprizone Administration

Two manners of cuprizone administration are used in animal experiments, both involving the oral route: (1) ingestion of a diet containing cuprizone [10], which has been employed in our study, and (2) administration via gavage [12]. These two administration ways lead to similar demyelination patterns; they do not require sophisticated equipment or surgical procedures, which makes the drug administration easily reproducible. In a recent study in mice, the demyelinating effectiveness of cuprizone has been confirmed and well-documented by Luxol fast blue/Cresyl violet staining, the immunolabeling of oligodendrocyte progenitors, and immunofluorescence [56]. These authors applied cuprizone in mice; however, the description of the cuprizone administration seemed confusing to us. In one paragraph of their article (Section 4.1), the authors state that “The demyelination group consisted of 10 male mice subjected to a 5-week diet supplemented with 0.2% cuprizone.” In another paragraph (Section 4.2), they state that “Cuprizone (CAS: 370-81-0, Sigma Aldrich, Vienna, Austria) was administered with drinking water at a concentration of 0.2% (*w*/*v*).” Regardless of the correct vehicle for cuprizone (diet or water), the demyelinating effectiveness of cuprizone has been confirmed. It would be exciting to compare the oral route with parenteral administration. However, parenteral cuprizone administration has not been performed to date.

## 6. Conclusions

Our results indicate that the electrophysiological and glial cell immunoreactivity effects of cuprizone are attenuated by physical exercise. Exercise counteracted the cuprizone-induced changes in CSD propagation and the numbers of Iba1- and GFAP-containing cells but not those of MBP- and APC-containing cells. Our data demonstrated that regular physical exercise is associated with a potential neuroprotective effect, which raises the possibility of benefiting patients with demyelinating disorders via this non-pharmacological, low-cost intervention.

## Figures and Tables

**Table 1:** Description of the six groups of rats in this study according to the two exercise conditions (exercised or sedentary) and with three dietary treatment conditions (con = control diet; Cup-5w = cuprizone diet for 5 weeks; Cup-5w/Con-6w = switched, after 5 weeks in the cuprizone diet, to the control diet to allow for remyelination for 6 weeks). The number of animals per group is presented in parentheses.

Groups (n)	Exercise Condition	Dietary Treatment
1 (9)	Exercised	Con
2 (10)	Sedentary	Con
3 (11)	Exercised	Cup-5w
4 (10)	Sedentary	Cup-5w
5 (10)	Exercised	Cup-5w/Con-6w
6 (8)	Sedentary	Cup-5w/Con-6w

**Table 2:** Body weights (in g) of sedentary and exercised rats at postnatal days P30 and P60. Cuprizone-treated groups received a commercial diet containing 2 g/kg cuprizone from postnatal day 28 (P28) to postnatal day 62 (P62). Control groups received the same commercial diet but without cuprizone. Data are expressed as mean ± standard deviation. * = *p* < 0.05 compared with the corresponding control group. # = *p* < 0.05 compared with the respective sedentary group.

Groups	Weight (in g) at 30 Days (n)	Weight (in g) at 60 Days (n)
Control, sedentary	86.4 ± 14.3 (10)	240.2 ± 13.1 (9)
Control, exercised	87.4 ± 12.3 (9)	201.3 ± 19.5 (9) #
Cuprizone-treated, sedentary	56.8 ± 16.8 (10) *	181.3 ± 16.8 (9) *
Cuprizone-treated, exercised	56.1 ± 10.7 (11) *	151.7 ± 18.4 (9) *#

## Data Availability

The article provides the primary data underlying this study. Additional inquiries regarding data availability can be addressed to the corresponding author upon reasonable grounds.
